# Severe acute respiratory syndrome coronavirus-2 Alpha variant (B.1.1.7), original wild-type severe acute respiratory syndrome coronavirus 2, and cytomegalovirus co-infection in a young adult with acute lymphoblastic leukemia, case report, and review of the possible cytomegalovirus reactivation mechanisms

**DOI:** 10.1186/s13256-022-03750-8

**Published:** 2023-02-10

**Authors:** Ali Amanati, Mahdi Shahriari, Mohammad Reza Bordbar, Seyyed Bozorgmehr Hedayati, Mazyar Ziyaeyan, Marzieh Jamalidoust, Mehdi Kalani, Nahid Heydari Marandi

**Affiliations:** 1 Professor Alborzi Clinical Microbiology Research Center, Shiraz University of Medical Sciences, Shiraz, Iran; 2 The Hematology Research Center, Shiraz University of Medical Sciences, Shiraz, Iran; 3 Departments of Pediatrics, Shiraz University of Medical Sciences, Shiraz, Iran

**Keywords:** SARS-CoV-2 Alpha variant (B.1.1.7), Original wild-type SARS coronavirus 2 (Wuhan strain), Cytomegalovirus, Co-infection, Gastroenteritis

## Abstract

**Background:**

Like other viral infections, severe acute respiratory syndrome coronavirus-2 infection could affect different human body systems, including host immune responses. Three years after its pandemic, we learn more about this novel coronavirus. As we expected, different co-infections with various organisms, such as viruses, bacteria, and even fungi, have been reported. However, concurrent infection with two severe acute respiratory syndrome coronavirus-2 strains and cytomegalovirus is extremely unusual. We have only a rudimentary understanding of such co-infections and their long-term consequences for patients with cancer.

**Case presentation:**

An 18-year-old young Iranian adult with acute lymphoblastic leukemia presented with abdominal pain, diarrhea, nausea, and vomiting following a recent history of severe acute respiratory syndrome coronavirus-2 infection. The patient never experienced respiratory symptoms, and the chest imaging study was normal on admission. His primary laboratory investigation revealed prerenal azotemia and severe abnormal liver function tests (blood urea nitrogen 32 mg/dL, creatinine 1.75 mg/dL, prothrombin time 66 s, partial thromboplastin time 44.5 s, international normalized ratio 5.14, total bilirubin 2.9 mg/dL, and direct bilirubin 2.59 mg/dL). Cytomegalovirus disease was diagnosed by polymerase chain reaction in his blood and stool samples. The patient’s gastrointestinal signs and symptoms improved shortly after receiving intravenous ganciclovir treatment. His gastrointestinal symptoms continued intermittently for weeks despite maintenance valganciclovir prescription, necessitating frequent hospitalizations. The patient was complicated by the recurrence of gastrointestinal symptoms during the sixth hospitalization, even though he had no respiratory symptoms, and the nasopharyngeal test revealed severe acute respiratory syndrome coronavirus-2 Wuhan strain for the first time. Remdesivir and valganciclovir were administrated due to persistent enteritis and evidence of intestinal tissue invasion by severe acute respiratory syndrome coronavirus 2 and cytomegalovirus on multiple intestinal biopsies, which led to partial clinical responses. Cytomegalovirus and severe acute respiratory syndrome coronavirus-2 fecal shedding continued for more than 6 months despite repeated antiviral therapy, and the Wuhan and Alpha strains were also detected in his nasopharyngeal samples through repeated sampling (confirmed by four nasopharyngeal sampling and multiple stool specimens and several intestinal biopsies). Finally, during the Delta-variant (B.1.617.2) outbreak in Iran, the patient was admitted again with febrile neutropenia and decreased level of consciousness, necessitating respiratory support and mechanical ventilation. During the Delta-variant peak, the patient’s nasopharyngeal sample once more tested positive for severe acute respiratory syndrome coronavirus 2. The patient died a few days later from cardiopulmonary arrest.

**Conclusion:**

The coronavirus disease 2019 pandemic has encountered patients with cancer with critical diagnostic and treatment challenges. Patients who are immunocompromised may co-infect with multiple severe acute respiratory syndrome coronavirus-2 strains and cytomegalovirus, and even with timely diagnosis and treatment, the prognosis may be poor.

**Supplementary Information:**

The online version contains supplementary material available at 10.1186/s13256-022-03750-8.

## Introduction

Over time, since the onset of the coronavirus disease 2019 (COVID-19) pandemic, our knowledge regarding pathogenesis, different clinical aspects, diagnosis, treatment, and the various severe acute respiratory syndrome coronavirus 2 (SARS-CoV-2) complications has improved. Gastrointestinal (GI) involvement is now one of the well-known presentations of SARS-CoV-2 infection [1–5]. In addition to the common GI symptoms such as nausea, vomiting, diarrhea, and abdominal pain, other GI complications have been described, such as acute hepatitis [6], hemorrhagic colitis [7], appendicitis [8–10], pancreatitis [11–13], and dysentery [14]. GI involvement could be associated with prolonged viral shedding, and fecal specimens may remain positive for SARS-CoV-2 even 2 weeks after the nasopharynx clearance, especially in those with diarrhea [15, 16]. GI symptoms could be continued for up to 3 weeks [16, 17], while infected patients may have asymptomatic fecal shedding for a more extended period, even more than 8 weeks [15–18]. The clinical significance of prolonged fecal excretion of the virus is not well understood; however, affected patients may be implicated in viral transmission [18–20]. There are few reports on SARS-CoV-2 and cytomegalovirus (CMV) co-infection in the literature [21–24]. Our current knowledge need to improve due to the significant complication of such co-infections [23]. The odds of fatal COVID-19 outcomes are higher among immunocompromised patients, even those receiving primary vaccination [25]. Although, co-infection of SARS-CoV-2 with more common seasonal viral infections such influenza viruses, respiratory syncytial virus, or adenoviruses has been thoroughly investigated [26], more research should be done on less frequent viral co-infections. This report aims to represent a young adult male with acute lymphoblastic leukemia (ALL) who developed CMV enteritis concomitant with SARS-CoV-2 Alpha variant (B.1.1.7) and original wild-type (Wuhan strain) SARS-CoV-2 co-infection. We also discuss possible mechanisms of CMV reactivation during SARS-CoV-2 infection.

## Case presentation

### Clinical history

On 27 December 2020, an 18-year-old young Iranian adult with a history of ALL who had been undergoing maintenance chemotherapy for more than 18 months and was in remission was admitted to our hospital owing to abdominal pain, diarrhea, nausea, and vomiting. He has a history of contracting SARS-CoV-2 infection 1 month before admission and subsequently repeated hospitalization. The patient never developed respiratory symptoms and did not fulfill the criteria for remdesivir treatment on first-time detection of SARS-CoV-2 via reverse transcription polymerase chain reaction (RT–PCR) test. The patient chemotherapy regimen consists of monthly vincristine (1.5 mg/m^2^), prednisolone (40 mg/m^2^) 5 days per month, weekly methotrexate (10 mg/m^2^), and 6-mercaptopurine (50 mg) every night. He also received trimethoprim/sulfamethoxazole 400/80 mg twice per day/three times per week for *Pneumocystis jirovecii* prophylaxis and voriconazole (VCZ) 200 mg twice per day concurrent with his chemotherapy. He has a history of invasive pulmonary aspergillosis and pan-sinusitis (8 and 4 weeks before admission, respectively). He was hospitalized with severe GI signs and symptoms in another academic hospital (leading to intensive care unit admission) without clinical improvement, leading to self-discharge. On admission, he was febrile (tympanic temperature 38.8 °C). He suffered from nausea/vomiting, abdominal pain, diarrhea, oral aphthous lesions, skin rashes, excessive drooling, low appetite, weight loss, and decreased urine output. The results of his primary lab testing are summarized in Table 1.

**Table 1:** Laboratory findings of patient coinfected with SARS-CoV-2/CMV on admission and during his admission course

	On admission	Day 1	Day 2	Day 3	Day 4	Day 5
WBC/mm^3^	15,370	20,580	15,630		15,630	
Hb (g/dL)	10.6	9.6	7.8		7.8	
Plt/mm^3^	80,000	140,000	186,000		186,000	
ESR (mm/h)	27					
CRP (mg/L)	19					
PT (seconds)	66	14.9	14.5			
PTT (seconds)	44.5	29.4	28.9			
INR	5.14	1.03	1			
BUN (mg/dL)	32	30	16	7		
Cr (mg/dL)	1.75	1.55	1.1	0.96		
Na	122	133	132			135
K	2.8	3	3			3.3
Ca	8.2	8.9				7.9
Mg	2.05	2.19				1.2
Uric acid	9	9.2				3.3
AST	39	41				48
ALT	51	66				42
T. Bil. (mg/dL)	2.9	3.47				2.65
D. Bil. (mg/dL)	2.59	2.71				1.83
Alb (mg/dL)	3.5	4.2				3.2
P	3.9	2.1				1.6

WBC, white blood cell; Hb, hemoglobin; Plt, platelet; ESR, erythrocyte sedimentation rate; CRP, C-reactive protein; PT, prothrombin time; PTT, partial thromboplastin time; INR, international normalized ratio; BUN, blood urea nitrogen; Cr, creatinine; Na, sodium; K, potassium; Ca, calcium; Mg, magnesium; UA, uric acid; AST, aspartate aminotransferase; ALT, alanine aminotransferase; T. Bil, total bilirubin; D. Bil, direct bilirubin; Alb, albumin; P, phosphor

On the basis of presenting signs and symptoms, severe abnormal liver function tests, and prerenal azotemia, we considered several differential diagnoses, including sepsis, drug–drug interactions or toxicities [methotrexate (MTX), vincristine (VNC), VCZ], SARS-CoV-2-induced gastroenteritis, biliary obstruction, portal venous thrombosis secondary to SARS-CoV-2 infection or VNC-induced veno-occlusive disease (VOD), and, finally, transfusion-associated graft-versus-host disease (TA-GVHD), given the history of the repeated transfusion (especially non-irradiated blood transfusion). Accordingly, he was worked up for drug–drug interactions. No significant interaction was found except VCZ/VNC. VCZ was withheld and VCZ serum levels were checked for three consecutive days, and all were within therapeutic or subtherapeutic ranges, 0.329 µg/mL, 0.241 µg/mL, and 0.526 µg/mL, respectively. MTX serum level also was checked, which was not in the toxic range (< 0.1 µmol/L), CYP2C19A polymorphism was also checked, which was compatible with homozygote CYP2C19*17 alleles (ultrarapid metabolizer phenotype). Serum galactomannan was negative on two occasions [0.3 and 0.2 optical density index (ODI)]. Given the prolonged prothrombin time (PT), partial thromboplastin time (PTT), and international normalized ratio (INR), a mixing test was requested. The mixing test corrected PT, PTT, and INR values in about 2 h. Factor assay was also performed, which showed decreased factor II and X levels, with acceptable factor V levels. On abdominopelvic ultrasonographic (US) study, the liver was normal in size with diffusely increased parenchymal echogenicity compatible with fatty liver change grade 2. Color Doppler sonography (CDS) of mesenteric and portal veins was done to investigate chemotherapy-induced VOD, which was normal.

When leukemia was diagnosed, the patient’s primary investigations revealed that he was CMV seropositive. Accordingly, a CMV blood PCR test was performed to check for CMV reactivation. The high viral load [98,500 copies/mL; cycle threshold (CT) 29] was suggestive of a significant CMV viremia. Stool was assessed for CMV by PCR because of hepatic impairment (abnormal liver function tests) and GI signs and symptoms. A fecal sample showed extremely high viral shedding (295,480,000 copies/mL; CT: 25). So, the patient was investigated for the possibility of disseminated CMV disease, including CMV encephalitis, despite having no central nervous system (CNS) signs or symptoms due to concurrent CMV viremia and CMV enteritis. CMV was not detected by PCR in CSF and urine specimens. We also investigated SARS-CoV-2 nasopharyngeal and stool PCR.

SARS-CoV-2 nasopharyngeal and stool RT–PCR also were positive (CT 31 and 24, respectively) for the Alpha strain. B.1.1.7 (Alpha), B.1.351 (Beta), and P1 (Gamma) variant’s single-nucleotide deletions were investigated for SARS-CoV-2 by a multiplex PCR using type-specific probes described by Vogels *et al.* (Additional file 1, Molecular Analysis for SARS-CoV-2 Variants Identification) [27].

**Fig. 1:**
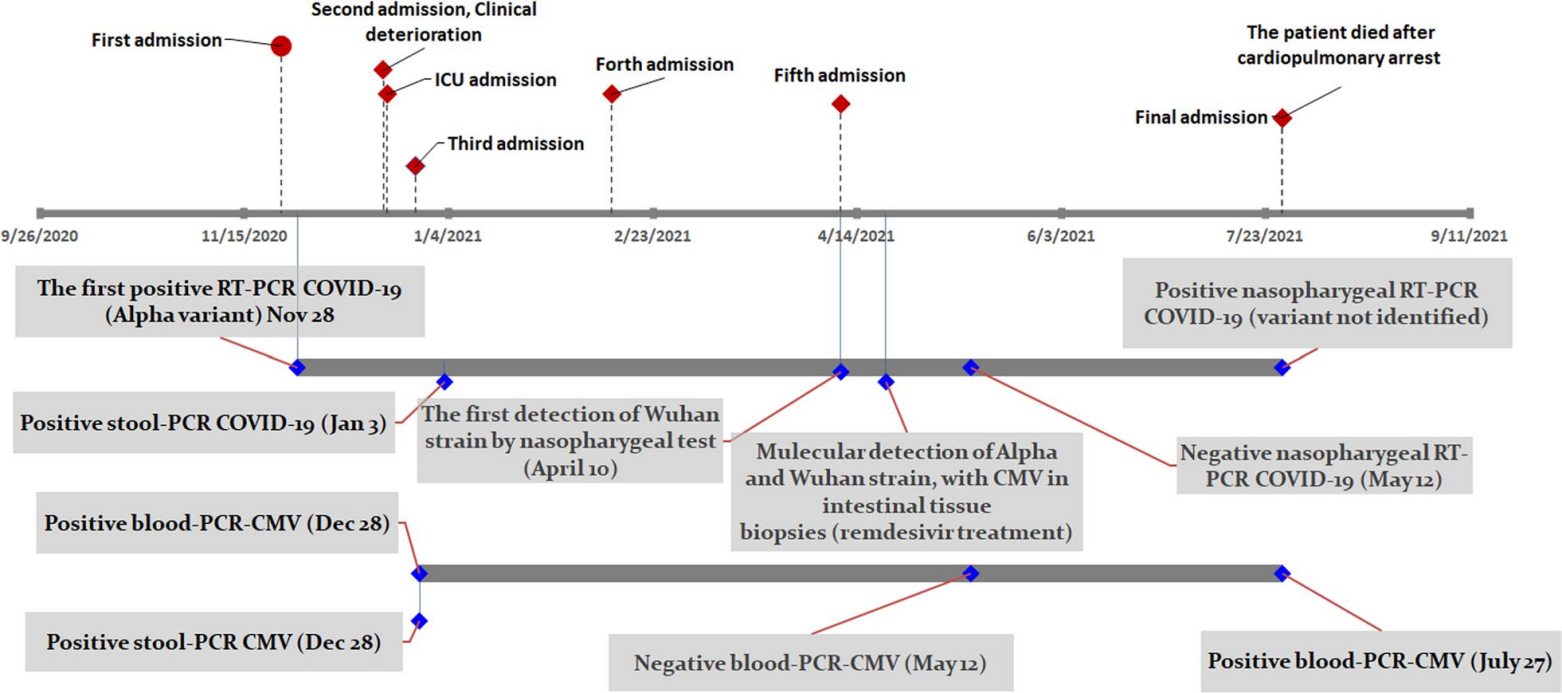
The patient’s viral markers status summary during repeated admissions (final admission not shown, see text for more information)

A summary of viral marker test results is presented in Table 1. The patient’s clinical course timeline and key clinical events during repeated hospitalization are shown in Fig. 1.

**Fig. 2:**
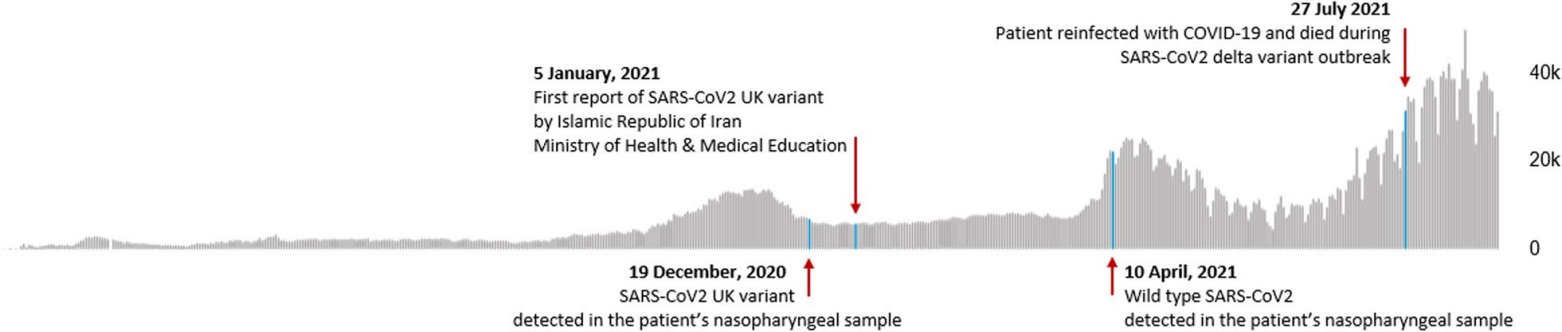
The patient’s SARS-CoV-2 PCR status compared with the national data of peaks of transmission in Iran. Data extracted from the WHO website last updated on 30 August 2021 (available at https://covid19.who.int/region/emro/country/ir) and Iran Ministry of Health and Medical Education website (available at https://behdasht.gov.ir/). The Alpha variant (B.1.1.7) was first sequenced in September 2020 in the UK and subsequently spread globally (https://assets.publishing.service.gov.uk/government/uploads/system/uploads/attachment_data/file/959438/Technical_Briefing_VOC_SH_NJL2_SH2.pdf)

The results of his virologic investigation are summarized in Table 2. Figure 2 also illustrates the patient’s SARS-CoV-2 status compared with the national data of transmission peaks.

**Table 2:** Virological characteristics of different clinical samples obtained from a young adult patient with acute lymphoblastic leukemia (ALL) during his inpatient and outpatient monitoring

Date	Sample type	Test	Result	Cycle threshold	Variant
19 December 2020	Nasopharyngeal	SARS-CoV-2	Positive	31	Alpha variant (B.1.1.7)*
28 December 2020	Stool	CMV	Positive	25	
28 December 2020	Blood	CMV	Positive	29	
30 December 2020	Urine	CMV	Negative	─	
30 December 2020	CSF	CMV	Negative	─	
3 January 2021	Stool	CMV	Positive	31.5	
3 January 2021	Blood	CMV	Positive	29	
4 January 2021	Stool	SARS-CoV-2	Positive	34	
19 January 2021	Stool	SARS-CoV-2	Negative	─	
19 January 2021	Stool	CMV	Negative	─	
20 January 2021	Blood	CMV	Positive	35	
13 February 2021	Blood	CMV	Positive	36	
14 February 2021	Stool	CMV	Negative	─	
14 February 2021	Stool	SARS-CoV-2	Negative	─	
15 February 2021	Blood	CMV	Negative	─	
16 February 2021	Stool	CMV	Negative	─	
17 March 2021	Nasopharyngeal	SARS-CoV-2	Positive	29	Alpha variant (B.1.1.7)
24 March 2021	Blood	CMV	Negative	─	
24 March 2021	Stool	CMV	Positive	32	
24 March 2021	Stool	SARS-CoV-2	Positive	33	
24 March 2021	Nasopharyngeal	SARS-CoV-2	Positive	21	Alpha variant (B.1.1.7)
10 April 2021	Nasopharyngeal	SARS-CoV-2	Positive	28	Wuhan strain
10 April 2021	Blood	CMV	Positive	34	
11 April 2021	Stool	CMV	Positive	35	
11 April 2021	Stool	SARS-CoV-2	Negative	─	
21 April 2021	Ileum (1)	CMV	Positive	30	
21 April 2021	Ileum (1)	SARS-CoV-2	Positive	28	Wuhan strain
21 April 2021	Right colon (2)	CMV	Positive	25	
21 April 2021	Right colon (2)	SARS-CoV-2	Positive	28	Alpha variant (B.1.1.7)
21 April 2021	Rectum (3)	CMV	Positive	29	
21 April 2021	Rectum (3)	SARS-CoV-2	Positive	42	Alpha variant (B.1.1.7)
10 May 2021	Blood	CMV	Negative	─	
10 May 2021	Stool	CMV	Positive	35	
10 May 2021	Stool	SARS-CoV-2	Negative	─	
12 May 2021	Nasopharyngeal	SARS-CoV-2	Negative	─	
27 July 2021	Nasopharyngeal	SARS-CoV-2	Positive	29	Delta variant?**
27 July 2021	Blood	CMV	Positive	31	
27 July 2021	Stool	CMV	Positive	31.6	

CMV, cytomegalovirus; *UK variant: SARS-CoV-2 B.1.1.7 variant; **The last nasopharyngeal SARS-CoV-2 RT-PCR was done during Delta-variant outbreak in Shiraz, while more than 80% of our cases related to Delta variant. Unfortunately, the patient died, and the variant investigation was not performed for the last specimen

The patient was treated with intravenous ganciclovir (5 mg/kg/dose Q12 hours as a loading dose) on 29 December 2020 once blood and stool CMV PCR confirmed CMV reactivation. Intragastrointestinal CMV replication was suppressed significantly after 1 week of ganciclovir treatment (stool PCR 20,800 copies/mL with CT 31.5). Blood viral load also decreased gradually up to the low level of detection (97,850, 1200, and 560 copies/mL) on repeated testing. Stool CMV PCR was negative (nondetectable) on follow-up testing in the sixth and seventh weeks of treatment. The patient’s treatment course was continued with oral valganciclovir 450 mg Q12 hours for 4 weeks. He had, however, experienced intermittent GI symptoms for several weeks following that, leading to recurrent hospitalizations. He received a short course of intravenous rehydration therapy (3–7 days) and supplementary electrolytes for correcting hypokalemia and hyponatremia in each admission. On 21 April 2021, a diagnostic rectosigmoidoscopy and colonoscopy were carried out, and several biopsies from the right colon, terminal ileum, and rectum were taken. No macroscopic or microscopic abnormality was detected during the operation and immunohistopathologic evaluation. It should be noted that diagnostic rectosigmoidoscopy and colonoscopy were postponed several times due to severe coagulopathy, profound neutropenia, and thrombocytopenia, in addition to the patient’s self-discharge events from the hospital.

**Fig. 3:**
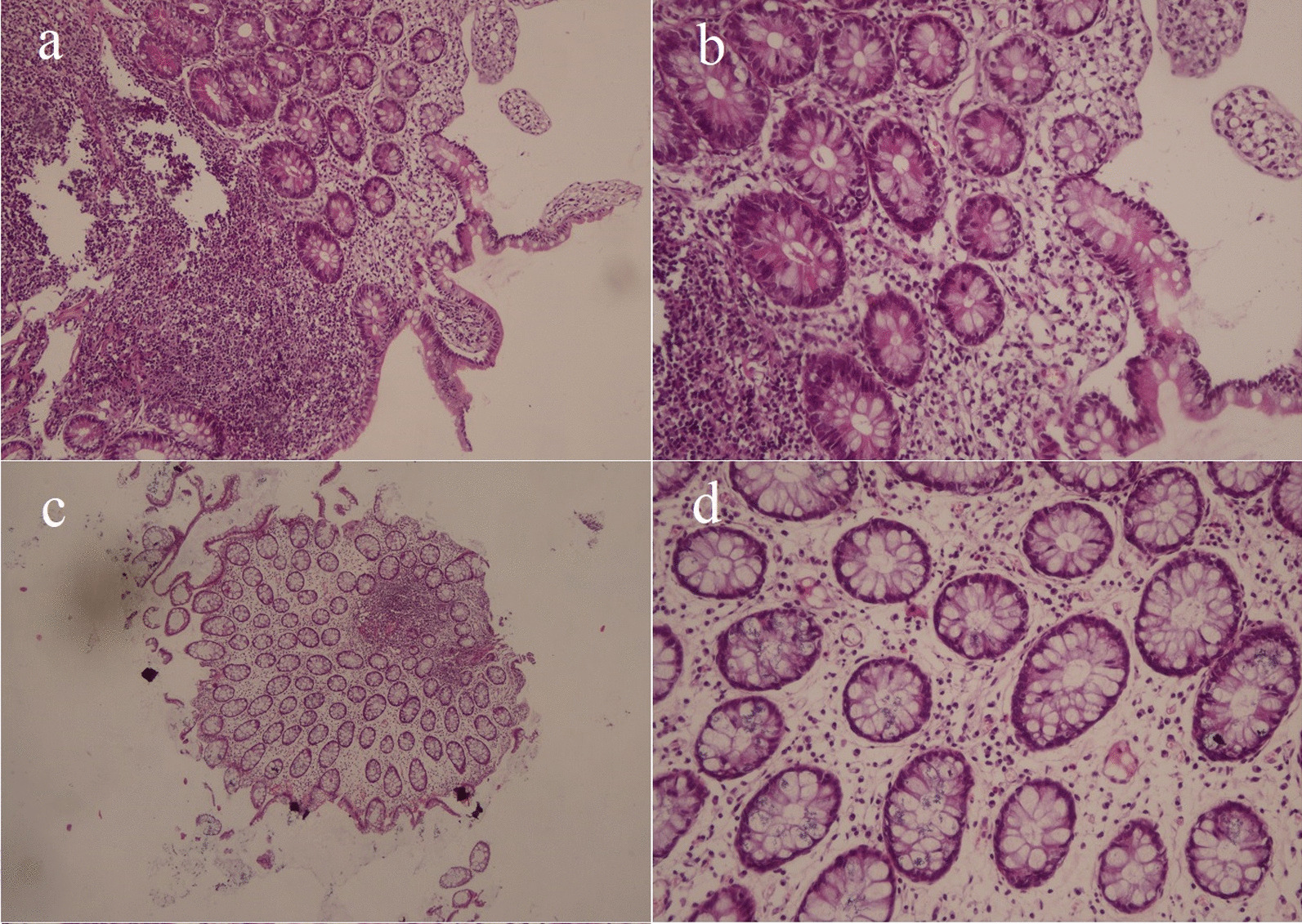
**a** Low-power view of the terminal ileum with normal villi without ileitis [hematoxylin and eosin (H&E) ×40]; **b** higher-power view of the same picture in Fig. 1. No inflammation or endothelial abnormality is seen (H&E ×200); **c** low-power view of colon mucosa biopsy shows normal-looking gland and surface epithelium. No colitis is present. A benign-looking lymphoid nodule is seen (H&E ×40); **d** higher-power view of the colon mucosa. No colitis is present. No abnormality of endothelium is seen, which is not indicative of CMV infection. Only scattered plasma cells and lymphocytes are present

CMV was detected by PCR in all tissue samples. SARS-CoV-2 Alpha variant (B.1.1.7) and original wild-type SARS-CoV-2 were also found in all three specimens. No specific CMV-cytopathic changes were reported on immunohistopathologic examination (Fig. 3a–d). The SARS-CoV-2 nasopharyngeal test was also positive for the *Wuhan* strain (CT 28). On 8 May 2021, the patient was treated with intravenous remdesivir (200 mg as a loading dose and 100 mg daily for 4 days) despite having no respiratory signs or symptoms. All GI manifestations resolved a few days after antiviral treatment, and he was encouraged to undergo close follow-up monitoring.

### Outcome and follow-up

**Fig. 4:**
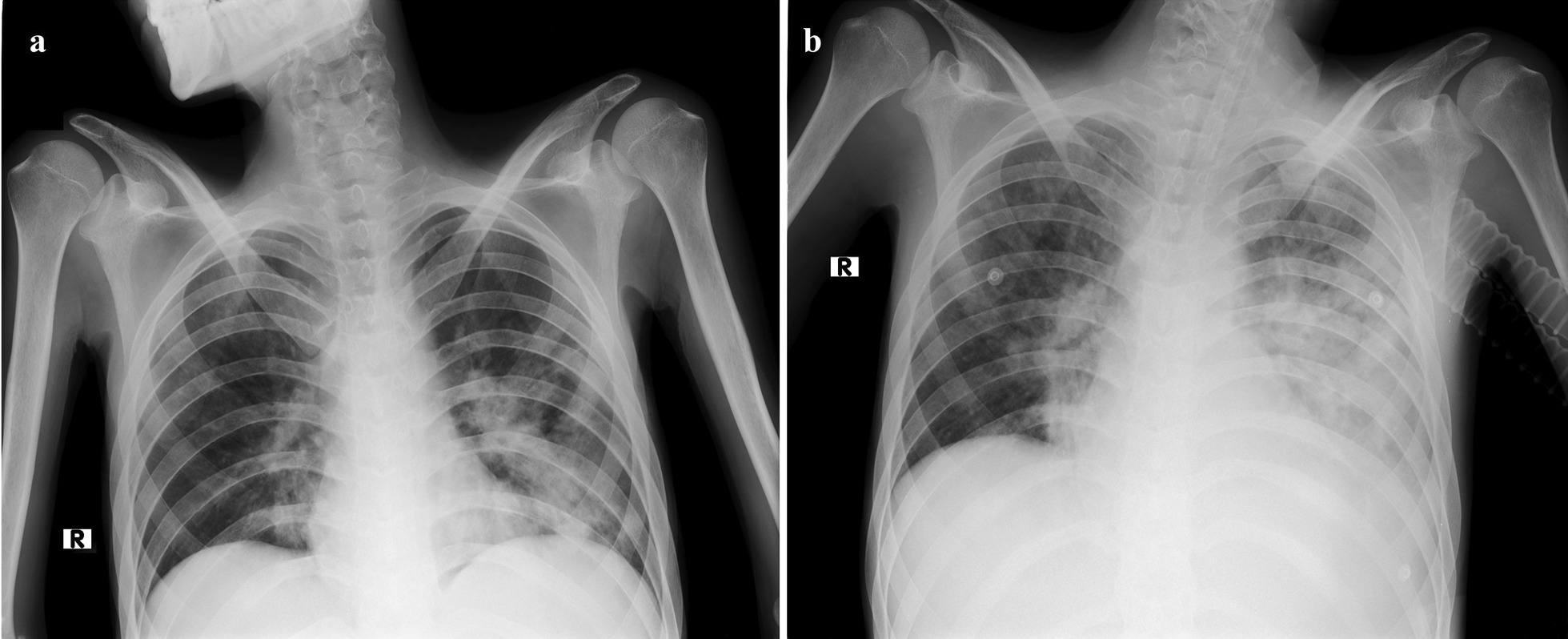
**a**, **b** The patient’s chest X-ray on his final admission leading to death. **a** Bilateral ground-glass opacities (more prominent in the left lung) on admission day; **b** rapid progressive parenchymal involvement of both lungs 4 days later

Finally, the patient was admitted again on 27 July, with febrile neutropenia and decreased level of consciousness necessitating respiratory support and mechanical ventilation. The patient’s nasopharyngeal specimen tested positive again, and blood and stool CMV PCR also were positive (30,500 and 19,700 copies/mL, respectively). The patient’s chest X-rays revealed bilateral multifocal parenchymal involvement (Fig. 4). The patient died after cardiopulmonary arrest on 27 July 2021.

**Fig. 5:**
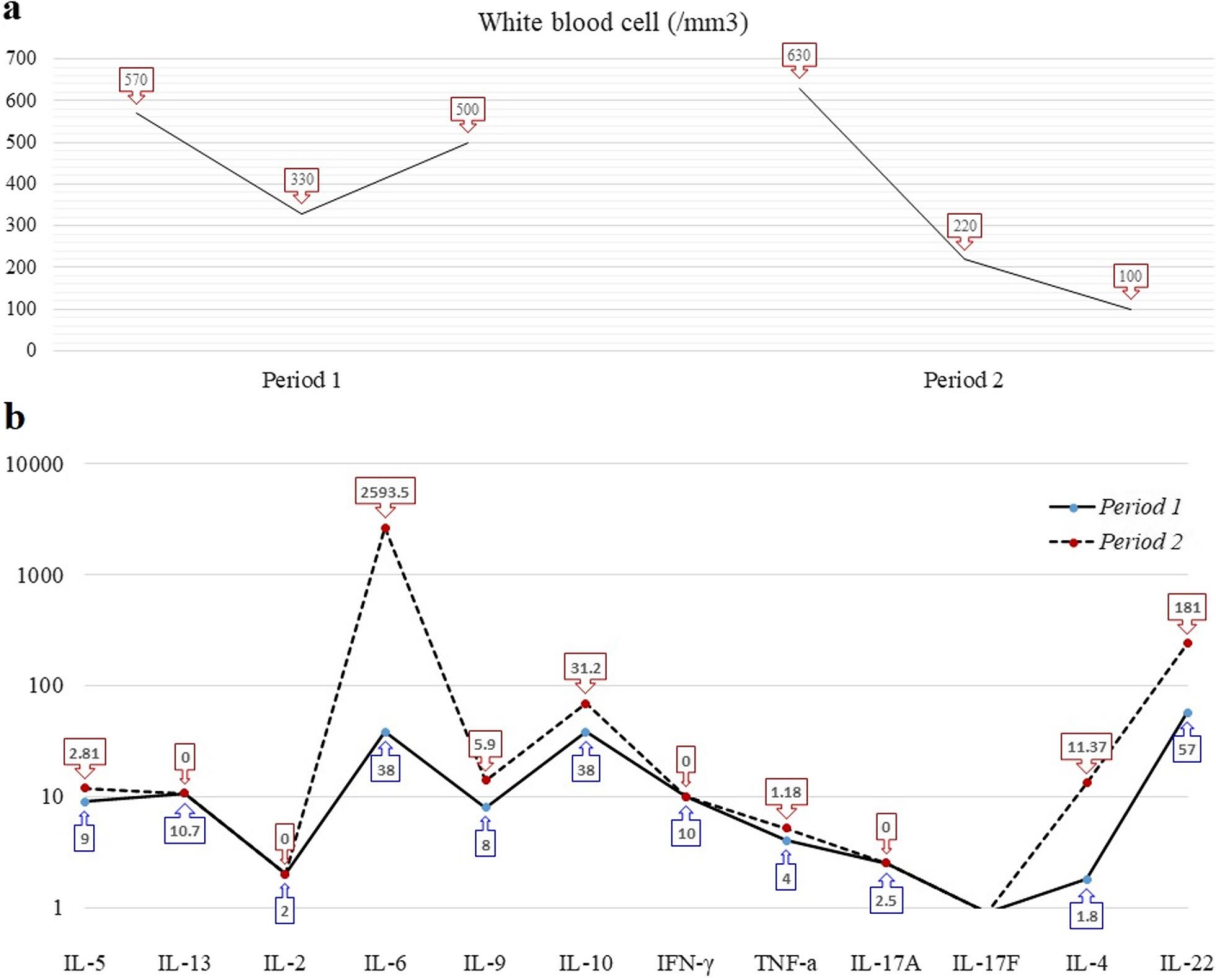
**a**, **b** The graphs depict variations in the white blood cell counts and immunological factors in the last two periods of patient hospitalization (period 1 and period 2, respectively). Period 2 represents the day before the patient died. **a** White blood cell counts during the last 3 days of each hospitalization period; **b** the patient’s cytokine assay during period 1 and period 2

We also perform immunological workups during the two periods of patient admissions, as shown in Fig. 5 (including the last two admissions where the second was 1 day before the patient died). The patient experienced severe neutropenia in both periods. Analysis of the serum cytokines revealed a similar pattern on two occasions; however, a substantial increase in IL-6 was found during the last admission (2593.5 versus 38 pg/mL) 1 day before the patient died. At this time, IL-17A and IL-17F decreased significantly to the nondetectable values. Analysis of the last lymphocyte immunophenotyping showed that the frequency of CD3^+^ CD4^+^ T cells was high (92%), whereas the levels of CD3^−^ CD16^+^ CD56^+^ lymphocytes (natural killer cells) as well as B lymphocytes (CD3^−^ CD19^+^ CD20^+^) were low (2% and 6%, respectively).

## Discussion

Here, we discuss a case with ALL that also had co-infection with CMV and two SARS-CoV-2 strains. The patient’s last bone marrow aspiration confirmed that he was in remission. The patient did not have other convincing reasons for the CMV reactivation, including the disease relapse, protocol change, anticancer agent dose intensification, or even nonadherence with chemotherapy treatment. On the other hand, CMV reactivation signs and symptoms (hepatic and gastrointestinal involvement) begin to develop immediately after COVID-19 infection. The patient was treated with ganciclovir due to CMV viremia and CMV enteritis even though he did not match the requirements for remdesivir treatment in the first detection of the SARS-CoV-2 RT-PCR test because he never developed respiratory symptoms. A nasopharyngeal test for the SARS-CoV-2 Wuhan strain showed up positive on the fifth admission. The patient was treated with remdesivir and valganciclovir for persistent enteritis and evidence of SARS-CoV-2 and CMV invasion of the intestinal tissue.

### SARS-CoV-2 PCR re-positivity and re-infection

Aside from the facts concerning the patient’s clinical condition during repeated admissions, there are some important points regarding interpreting the patient’s repeated positive viral markers.

A positive PCR test within 3 months of past COVID-19 infection in an asymptomatic individual with a CT value higher than 35 is usually considered a PCR re-positivity, and there is no need for respiratory isolation [28, 29]. However, all CT values were lower than 35 in our case during more than 6 months of follow-up (Table 2), which could be explained by the patient’s immunosuppressed status [30]. Accordingly, we considered respiratory precautions and isolation in his ambulatory visits and admissions. On the basis of available guidelines, those experiencing atypical clinical courses and having a history of exposure during outbreaks are suspected of re-infection [28]. So, according to prolonged PCR re-positivity with low CT values and also due to his immunosuppressed status, he was suspected of contracting a new mutant SARS-CoV-2 strain, and he was investigated for other variants of concern (VOCs), including B.1.351 (Beta) and P1 (Gamma).

Interestingly, the SARS-CoV-2 Wuhan strain was detected in the patient’s pharyngeal sample. The re-infection with SARS-CoV-2 Wuhan strain (CT value 28) is particularly intriguing, given that the patient’s pharyngeal specimen was positive about 16 days earlier with Alpha strain (CT value 21). It is unusual and unreasonable to document a new SARS-CoV-2 strain while the patient has recently had a positive pharyngeal test with another SARS-CoV-2 strain. It is worth noting that, unlike some bacterial strains, such as *Haemophilus influenza*, which can have pharyngeal colonization for months, documentation of viral infection (such as the flu) in the upper respiratory tract is evidence of acute infection. The simultaneous presence of Wuhan and Alpha SARS-CoV-2 strains (all CT values lower than 35; Table 2), in addition to CMV in the patient’s intestinal tissue samples, is also a thought-provoking finding in our patient.

### SARS-CoV-2 GI involvement

Our patient suffers from prolonged SARS-CoV-2 shedding confirmed by consecutive positive PCR results from his nasopharyngeal and stool specimens for about 6 months. Although prolonged viral shedding is a well-known phenomenon in immunocompromised hosts after COVID-19 infection, it has never been reported for such a long time [31, 32]. It should be noticed that the prolonged SARS-CoV-2 shedding in cancer patients is associated with poor prognosis and increased cancer-associated death and comorbidities [33], as we described in this report.

GI, hepatic, hypercoagulability state, and prerenal azotemia successfully improved within a few days after treatment. Similar clinical presentation of COVID-19 was our main challenge; however, as we discussed here, CMV reactivation is the most probable cause of multiorgan involvement in this case. The possible CMV reactivation mechanisms are also briefly discussed here on the basis of current data regarding CMV reactivation in critically ill patients.

Angiotensin-converting enzyme 2 (ACE2) is highly expressed in the lung cells, esophagus epithelial cells, and absorptive enterocytes of the ileum and colon [1]. As a result, GI involvement could occur during SARS-CoV-2 infection and worsen the patient’s outcome [15]. Diarrhea, nausea/vomiting, and abdominal pain are the most common signs and symptoms [16]. GI involvement may be the sole manifestation of the SARS-CoV-2 infection in about 20% of affected individuals [15]. The most common reported lab findings are elevated liver enzymes (mostly aspartate transaminase), elevated alkaline phosphatase, bilirubin, and abnormal PT values [34]. The median time for GI symptom presentation is about 4 days after infection, and may last for up to 2 weeks [34], although researchers found a shorter course in those with milder COVID phenotypes [15]. Nonspecific, moderate microvesicular steatosis, mild mixed lobular, focal/portal necrosis, and portal lymphocyte infiltration suggestive of hepatic vascular involvement could be seen on liver histology [35]. Fecal SARS-CoV-2 shedding may occur in about half of cases [34], which usually lasts longer than nasopharyngeal viral shedding (about 2 weeks or longer) [36]. GI manifestations rarely continued for more than 3 weeks [16]. Altogether, based on the natural history of SARS-CoV-2 infection, intermittent fecal viral shedding, PCR CT values higher than 30 in fecal specimens (in all tested samples), and the too late presentation, the patient’s signs and symptoms could not be attributed to COVID-19.

### Possible CMV reactivation mechanisms in the context of SARS-CoV-2 infection

Understanding the likely mechanism of CMV reactivation in SARS-CoV-2 infection is also a debatable issue with considerable importance.

CMV is a genetically diverse large virus with more than 200 open reading frames producing effector proteins, about one-quarter of them committed to CMV replication. It may potentially alter innate and adaptive host immune responses [37–40]. There is very little information regarding CMV reactivation mechanisms in the literature. Immediate early (IE) CMV gene, which is expressed by TNF-α stimulation, sepsis-induced immunosuppression, decreased IFN-γ T-cell responses, natural killer (NK) dysfunction, and impaired dendritic cell function all could be attributed to CMV reactivation after sepsis from a bacterial origin [41–46]. The mechanisms of CMV reactivation during viral infections are much less known. However, elevated TNF-α and cyclic-AMP resulting from pro-inflammatory prostaglandins and stress catecholamine stimulation may play a role [47]. Finally, the role of T-regulatory/T-helper 17 (Treg/Th17) imbalance has been described previously in the pathogenesis of cytomegalovirus reactivation [48].

**Fig. 6:**
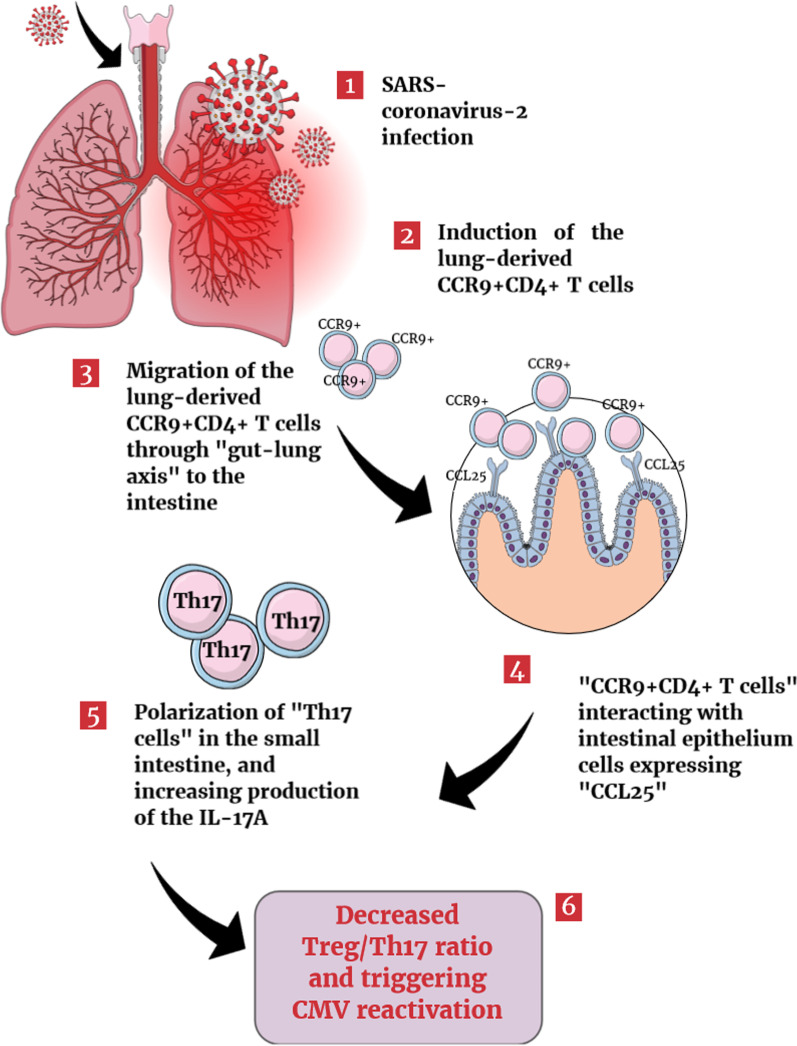
Suggested cytomegalovirus reactivation mechanism after SARS-CoV-2 infection [43–45]

On the basis of previous discussions, reactivation of cytomegalovirus may occur through previously known mechanisms in the context of COVID-19 infection. Gastrointestinal endothelial cells could be affected indirectly by lung infection due to immune regulation of the “gut–lung axis” in the absence of detectable SARS-CoV-2 in the stool of patients with COVID-19. Following SARS-CoV-2 lung infection, CD4^+^ T cells infiltrate the infected site and express CCR9 upon stimulation. After that, lung-derived CCR9^+^ CD4^+^ T cells migrate by the systemic circulation to the small intestine where epithelial cells express CCL25. Interaction between CCR9 and CCL25 is essential for recruiting effector T cells to the intestine [49]. Upon infiltration of CCR9^+^ CD4^+^ T cells, a disturbance occurs in the intestine flora, affecting the balance between Th17 and regulatory T cells, leading to the increased polarization of Th17 and, consequently, more IL-17A production. IL-17A recruits neutrophils into the intestine, which can cause intestinal immune damage [50]. As discussed earlier, Treg/Th17 balance is essential in the pathogenesis of cytomegalovirus infection. During CMV reactivation/replication, decreased Treg/Th17 ratio could be observed with decreased Treg-related factors and increased Th17-related factors (Fig. 6), while in latent infection Treg/Th17 ratio usually increased secondary to robust Treg responses [48]. Thus, mediators of host immune responses could be affected due to the SARS-CoV-2 leading to the CMV reactivation. However, the hypothesis will require additional investigation through thoughtful *in vitro* experiments.

A few considerations should be considered regarding the justification of the negative cytopathic effect in the histopathologic examination. First, sample biopsies may be taken from the unaffected areas. Second, the likelihood of identifying a cytopathic effect in a critically symptomatic patient with severe diarrhea may be minimal due to the significant mucosal shedding of the injured endothelium. Finally, previous effective antiviral treatment may playing an important role. Despite the absence of the expected cytopathic effect, the extremely high fecal viral load shows extensive GI tract involvement when pathological investigation results are inconclusive. Besides, high-level CMV fecal shedding during CMV viremia without invasive intestinal disease may occur in immunocompromised patients as described previously [51, 52].

### Cytokine and biomarker investigation during CMV and SARS-CoV-2 co-infection in critically ill patient with cancer

We observed that, in contrast to the reduced leukocyte count, the levels of IL-6, which is essential for Th17 differentiation [53], and IL-22 produced by Th22 and Th17 [54] increased significantly in the final stages; however, IL-17A and IL-17F secreted by Th17 had the lowest levels. Regarding T-cell exhaustion in viral infections such as CMV and SARS-CoV-2 [55, 56], it seems that, besides leukopenia, higher IL-6 levels may also affect T-cell exhaustion and secretion of IL-17 by Th17 cells. So, the Treg/Th17 balance is disturbed at the terminal stages of the CMV/SARS-CoV-2 co-infection. To confirm bone marrow (BM) remission during the last admission, the patient underwent a trephine bone marrow aspiration/biopsy once more.

CMV co-infection may be fatal in high-risk patients such as transplant recipients [23]. Prolonged intermittent GI symptoms in leukemic individuals during the COVID-19 pandemic may mimic more severe and prevalent GI illnesses such as GVHD or irritable bowel syndrome (IBS), especially in the setting of CMV co-infection.

## Conclusion

Patients with cancer have encountered critical diagnostic and treatment challenges as a result of the COVID-19 pandemic. Human immune responses may be affected by SARS-CoV-2/CMV co-infection. Such reports may help us with current virus–virus interactions during the COVID-19 pandemic. However, further research is needed to confirm CMV reactivation/replication mechanisms in the context of SARS-CoV-2 infection.

## Supplementary Information

**Additional file 1.** Molecular Analysis for SARS-CoV-2 Variants Identification.

## Data Availability

All data generated or analyzed during this study are included in this article.

## Abbreviations

SARS-CoV-2: Severe acute respiratory syndrome coronavirus-2
CMV: Cytomegalovirus
ALL: Acute lymphoblastic leukemia
PCR: Polymerase chain reaction
GI: Gastrointestinal
COVID-19: Coronavirus disease 2019
MTX: Methotrexate
VNC: Vincristine
VCZ: Voriconazole
aGVHD: Acute graft-versus-host disease
VOD: Veno-occlusive disease
ODI: Optical density index
PT: Prothrombin time
PTT: Partial thromboplastin time
INR: International normalized ratio
CSF: Cerebrospinal fluid
CT: Cycle threshold
US: Ultrasonographic
CDS: Color Doppler sonography
ACE2: Angiotensin-converting enzyme 2
NK: Natural killer
Treg: T-regulatory
Th17: T-helper 17
