# Supplementary material for: Severe acute respiratory syndrome coronavirus-2 Alpha variant (B.1.1.7), original wild-type severe acute respiratory syndrome coronavirus 2, and cytomegalovirus co-infection in a young adult with acute lymphoblastic leukemia, case report, and review of the possible cytomegalovirus reactivation mechanisms
Source: J Med Case Rep. 2023 Feb 10;17:66. doi: 10.1186/s13256-022-03750-8 (PMC9913040; doi:10.1186/s13256-022-03750-8)
Supplement: Supplementary file 1 — Additional file 1. Molecular Analysis for SARS-CoV-2 Variants Identification. [file 13256_2022_3750_MOESM1_ESM.docx]

**Molecular Analysis for SARS-CoV-2 Variants ‎Identification‎ ‎**

Genotyping of SARS-CoV 2 was performed by using the information provided in the reference number 26 [Vogels et al.]. In the first step, for detection of SARS-CoV-2, we use generic general primers and probes, and after getting positive results we have done genotyping PCR. In this case, B.1.1.7 was expected and we determined 69/70 deletion on spike gene accompanying with ORF1a 3675-3677 deletion. Because of the persistence of these two deletions, we consider the results as B.1.1.7. Of course, any measurements regarding contamination were considered.
